# Injury mechanism of COVID-19–induced cardiac complications

**DOI:** 10.1097/CP9.0000000000000055

**Published:** 2023-06-23

**Authors:** Ling Leng, Xiu-Wu Bian

**Affiliations:** 1 Stem Cell and Regenerative Medicine Lab, Department of Medical Science Research Center, State Key Laboratory of Complex Severe and Rare Diseases, Translational Medicine Center, Peking Union Medical College Hospital, Peking Union Medical College and Chinese Academy of Medical Sciences, Beijing 100730, China.; 2 Institute of Pathology and Southwest Cancer Center, Southwest Hospital, Third Military Medical University (Army Medical University), and Key Laboratory of Tumor Immunopathology, Ministry of Education of China, Chongqing 400038, China.; 3 Department of Pathology, the First Hospital Affiliated to University of Science and Technology of China (USTC), and Intelligent Pathology Institute, Division of Life Sciences and Medicine, USTC, Hefei 230036, China.

**Keywords:** COVID-19, Cardiovascular diseases, Molecular pathology, SARS-CoV-2

## Abstract

Heart dysfunction is one of the most life-threatening organ dysfunctions caused by coronavirus disease 2019 (COVID-19). Myocardial or cardiovascular damage is the most common extrapulmonary organ complication in critically ill patients. Understanding the pathogenesis and pathological characteristics of myocardial and vascular injury is important for improving clinical diagnosis and treatment approach. Herein, the mechanism of direct damage caused by severe acute respiratory syndrome coronavirus 2 to the heart and secondary damage caused by virus-driven inflammation was reviewed. The pathological mechanism of ischemia and hypoxia due to microthrombosis and inflammatory injury as well as the injury mechanism of tissue inflammation and single myocardial cell necrosis triggered by the viral infection of pericytes or macrophages, hypoxia, and energy metabolism disorders were described. The latter can provide a novel diagnosis, treatment, and investigation strategy for heart dysfunctions caused by COVID-19 or the Omicron variant.

## INTRODUCTION

There have been at least six million deaths led by the novel coronavirus disease 2019 (COVID-19) on a global scale^[1]^. Heart complications are among the most severe manifestations in patients with COVID-19^[2–4]^. As the incidence of myocarditis and cardiovascular diseases due to COVID-19 continuously increases, the effect of COVID-19 on heart tissues has garnered considerable attention. The cardiovascular system affected by COVID-19 has complications including myocardial injury, myocarditis, myocardial infarction, heart failure, arrhythmia, and venous thromboembolic events^[5]^. In view of the essential role of heart disease symptoms in patients with COVID-19, it is essential to identify the cardiac injury mechanism of COVID-19. Previously, several mechanisms have been postulated to explain COVID-19–associated cardiac injury, including direct myocardial injury mediated via angiotensin converting enzyme 2 (ACE2), immune dysregulation mediated by cytokine storm, hypoxia from imbalance in oxygen supply, cardiotoxicity of antiviral drugs as well as demand mediated by ischemia^[6–9]^. Descriptions regarding the pathological presentation of COVID-19 cardiac injury was predominantly obtained from autopsy-based literature. Cardiac abnormalities have been discovered in the gross pathology or histologic findings in approximately all of the cases. In these cases, the average age of the deceased was 69 years, and male cases account for more than half of all cases^[10]^.

## MOLECULAR PATHOLOGY REVEALS THE PRESENCE OF SARS-CoV-2 IN THE HUMAN HEART

Currently, researchers utilize reverse transcription polymerase chain reaction (RT-PCR), *in situ* hybridization, electron microscopy, and other methodologies to detect and localize severe acute respiratory syndrome coronavirus 2 (SARS-CoV-2) infections in the heart. Furthermore, proteome technology is used to obtain a panoramic view of molecular changes in cardiac proteins in patients with COVID-19^[11–15]^. To verify whether the virus can self-replicate in the heart, Bulfamante *et al.*^[16]^ employed digital PCR technology to detect viral RNA in the total RNA extracts from heart tissue samples and detected viral RNA in all six COVID-19 heart specimens, although not in the healthy controls. The copy number of the SARS-CoV-2 RNA molecule is 4.44–5.33 log10 (copies/mL). Lindner *et al.*^[12,13]^ used RNA range *in situ* hybridization to detect SARS-CoV-2 viral RNA minus strands in autopsy heart tissues and reported that the virus was detected in 61.5% patients while a high viral load of >1,000 copies/μg RNA was identified in 41.0% patients, in whom the expression levels of six proinflammatory factors (tumor necrosis factor α [TNF-α], interferon γ [IFN-γ], C–C motif ligand 5 [CCL5], interleukin [IL]-6, IL-8, and IL-18) were upregulated compared with that in the patients with low-virus copies. However, the degree and levels of myocardial leukocyte infiltration in patients with high-virus copies were not significantly different from those of the other patients^[16]^.

Brook *et al.*^[17]^ performed *in situ* hybridization experiments on tissue samples obtained via minimally invasive autopsy and about 10% of the patients were reported with a low viral load (not more than 120 copies/μg total RNA). Hanley *et al.*^[18]^ collected fresh cardiac tissue from autopsy samples and quantified viral load via quantitative real-time PCR (qRT-PCR), targeting the viral E gene, and reported higher viral loads in 2 of 5 patients. Lindner *et al.*^[12]^ detected SARS-CoV-2 viral particles in the heart tissue obtained from 24 of 39 (59%) consecutive autopsies. Subsequently, additional studies have reported SARS-CoV-2 copies in heart tissues^[12,19,20]^, further suggesting that SARS-CoV-2 can infect the heart tissue directly. The abovementioned studies demonstrate that the virus can self-replicate in the heart; however, the results based on minimally invasive autopsies may considerably vary from those of other studies owing to the heterogeneity of the collected materials.

To examine the localization of SARS-CoV-2 in the infected heart, Bois *et al.*^[14]^ performed immunohistochemistry with SARS-CoV-2 nucleocapsid–based antibodies. The results revealed focal and nonspecific staining in all the evaluated samples, with three patients exhibiting arterial or capillary endothelial cells staining and one patient exhibiting endocardium and macrophages staining. Bradley *et al.*^[21]^ utilized SARS-CoV-2 immunohistochemistry using a monoclonal antibody against the spike protein; nonetheless, the immunohistochemical results of SARS-CoV-2 S protein in patients with myocarditis were negative.

Fox *et al.*^[22]^ detected particles consistent with the SARS-CoV-2 in cardiac endothelial cells but not in adjacent cardiomyocytes (CMs) using electron microscopy. In that study, CD8^+^ and CD4^+^ lymphocytes were primarily identified in or near small vessels. Schurink *et al.*^[19]^ using two different antibodies against SARS-CoV-2 nucleocapsid proteins demonstrated positive immunohistochemical staining of CMs in 27% patients. Bulfamante *et al.*^[16]^ utilized immunohistochemistry to identify SARS-CoV-2 nuclear and spike proteins in left ventricular heart samples, which were positive for lipofuscin expression, predominantly in CMs, and confirmed this result via the immunofluorescence staining of viral nucleolin and sarcomeric α-actin (αSARS)-positive cells. This finding was verified via immunofluorescence costaining of viral nucleoprotein with αSARS-positive cells.

In the minimally invasive autopsy of 11 children infected with COVID-19, Dolhnikoff *et al.*^[23]^ detected viral particles in CMs, capillary endothelial cells, endocardial endothelial cells, macrophages, neutrophils, and fibroblasts in the patient’s heart. Gauchotte *et al.*^[24]^ performed immunohistochemical assessment using anti-SARS-CoV-2 nucleocapsid protein antibodies and demonstrated intense multifocal granular cytoplasmic staining in CMs. Moreover, in heart tissue, immunohistochemistry using anti-CD163 antibodies revealed numerous stromal macrophages, while anti-CD20, anti-CD3, anti-CD4, and anti-CD8 staining revealed that the vast majority of lymphocytes were CD3^+^, CD8^+^, and T-cell intracytoplasmic antigen (TIA)-1 cytotoxic T lymphocytes. Although, Rapkiewicz *et al.*^[25]^ did not discover any viral inclusion bodies via cardiac electron microscopy, they reported platelet-fibrin microthrombi and apparent megakaryocytes in the heart microvasculature.

## SARS-CoV-2–INDUCED DIRECT MYOCARDIAL INJURY

Various pathological reports have identified SARS-CoV-2 viral proteins and genetic material in the CMs of patients with COVID-19^[16,26]^. Furthermore, *in vitro* studies using human induced pluripotent stem cells (hiPSC) and isolated adult CMs as well as *in vivo* experiments using animal models confirm that SARS-CoV-2 infection occurs in CMs^[27–30]^. Collectively, these data support the hypothesis that COVID-19–associated myocardial injury can be caused by the direct infection of CMs and cardiotoxic defects in SARS-CoV-2.

### Direct disruption of the sarcomere structures by SARS-CoV-2

The pathogenic mechanisms of SARS-CoV-2 are commonly considered multidimensional. Among these mechanisms, one is based on data suggesting that viral infection directly disrupt sarcomeres in CMs. Sarcomere disruption and muscle fiber loss in CMs have also been sporadically observed *in vitro* and *in vivo* studies with similar features^[16,20,27]^. Perez-Bermejo *et al.*^[31]^ discovered that myofibrillar fragments in the cytoplasm are produced by gradually dividing the sarcomeres into single sarcomeric units in the cytoplasm of hiPSC–CM with viruses infection time, indicating that SARS-CoV-2 infection exacerbated this nonspecific cytopathy. In addition, at the genetic level, genes encoding sarcomere structural proteins, myosin light chains, and linkers of nucleoskeleton and cytoskeleton complexes (a subset of important proteins that anchor the nucleus the actin cytoskeleton) were significantly reduced in infected hiPSC–CMs. Moreover, altered gene expression was correlated with energy production in hiPSC–CMs, which -CMs triggers a shift from mitochondrial oxidative phosphorylation to glycolytic metabolism^[29]^.

Researchers further assessed the contractile properties of hiPSC–CMs at the tissue level using three-dimensionally engineered heart tissue (3D-EHTs) and observed that 3D-EHTs infected with SARS-CoV-2 exhibited weaker contractile force compared with the mock control group^[29,32]^. In addition to the mechanical dysfunction, the SARS-CoV-2–infected CMs demonstrate impaired electrical function^[33]^. Marchiano *et al.*^[29]^ discovered that SARS-CoV-2 infection can result in abnormal generation and transmission of electrical signals from hiPSC–CMs and human embryonic stem cell–derived CMs (hESC–CMs), demonstrating reduced beating rate, peak amplitude of depolarization, and electrical conduction velocity. The formation of syncytia in SARS-CoV-2–infected cells and tissue has also been reported, which is relatively rare^[34]^. Coronaviruses commonly induce cell–cell fusion because of the S protein fusion properties and its ability to trigger virus–cell membrane fusion. Based on the above, these studies provide a new perspective regarding the pathogenic mechanisms of COVID-19–associated arrhythmias and validate the deleterious effects of the direct infection of CMs with SARS-CoV-2.

### Straight cardiac injury tightly associated with ACE2

SARS-CoV-2 infects host cells by attaching its surface spike protein to the human ACE2 receptor. Briefly, the spike protein is activated by transmembrane protease serine 2 (TMPRSS2), allowing the fusion of SARS-CoV-2 and host cellular membranes^[7,8,35]^. After binding, SARS-CoV-2 enters the cell, consequently altering the ACE2 pathway and leading to cardiac injury. Another study reported that ACE2-dependent SARS-CoV-2 infection can decrease myocardial ACE2 protein expression correlated with macrophage infiltration and myocardial damage^[36]^. Cells expressing high levels of ACE2, including CMs, are more susceptible to SARS-CoV-2 invasion, which subsequently leads to organ damage^[37]^. A single-cell sequencing study^[38]^ reported high expression levels of the viral ACE2 receptor in the CMs of normal hearts and that ACE2 expression is higher in the heart than in the lung of adults. In the absence of ACE2 in CMs, a crosstalk between angiotensin II (Ang II) and angiotensin-(1-7) (Ang- [1–7]) signaling may have more deleterious effects in the heart. The detrimental effect of ACE2 downregulation would inhibit the cardioprotective effects of Ang-(1–7), leading to increased TNF-α production^[39,40]^. Moreover, ACE2 expression was substantially increased in the CMs of cardiac patients (e.g., dilated cardiomyopathy, hypertrophic cardiomyopathy, non–COVID-19 myocarditis, aortic stenosis, and heart failure) compared with healthy controls^[41–43]^. This finding indicates that patients with COVID-19 infection and underlying cardiovascular diseases are more susceptible to myocardial injury^[44,45]^.

The downregulation of ACE2 may result from the activation of a disintegrin and metallopeptidase domain-17/tumor necrosis factor-alpha converting enzyme (ADAM-17/TACE) by SARS spike proteins that cleave and release ACE2 or endocytosis and the subsequent degradation of the ligand/receptor complex^[46,47]^. Type II transmembrane serine protease (TMPRSS2) is a protein implicated in the cleavage of numerous SARS-CoV-2 variants. TMPRSS2 degrades S protein into S1 and S2 subunits, exposing the receptor binding domain of the S1 subunit to facilitate the identification of and binding to the ACE2 receptor. Bailey *et al.*^[32]^ reported the inhibition of TMPRSS2 and observed no defect in hiPSC–CMs infection while endosomal cysteine protease inhibitors eliminated SARS-CoV-2 infection. These data indicate that endosomal-dependent proteases can compensate for S protein priming to facilitate SARS-CoV-2 infection in CMs. Nevertheless, Omicron, a variant of SARS-CoV-2, requires only ACE2 to invade cells and no TMPRSS2 mediation. This variant is enclosed in endosomal bubbles, which floats into the cells and bursts^[48]^. Thus, the specific role of Omicron in cardiac injury requires further clarification.

### Directly infection of the interstitial cells in heart by SARS-CoV-2

In some published cases, the viral particles of SARS-CoV-2 were not isolated within the CMs, but rather in the interstitial cells, including pericytes and macrophages. SARS-CoV-2–infected pericytes may play a role in capillary endothelial cell or microvascular injuries and individual cell necrosis^[12,22,49]^; nevertheless, SARS-CoV-2-macrophages infected can mediate both local and systemic responses to viral infection, which can also repair the complement, potentially causing the death of myocytes by activating apoptotic attack complexes^[50]^.

### COVID-19–associated myocarditis may differ from traditional viral myocarditis

**Figure 1.**
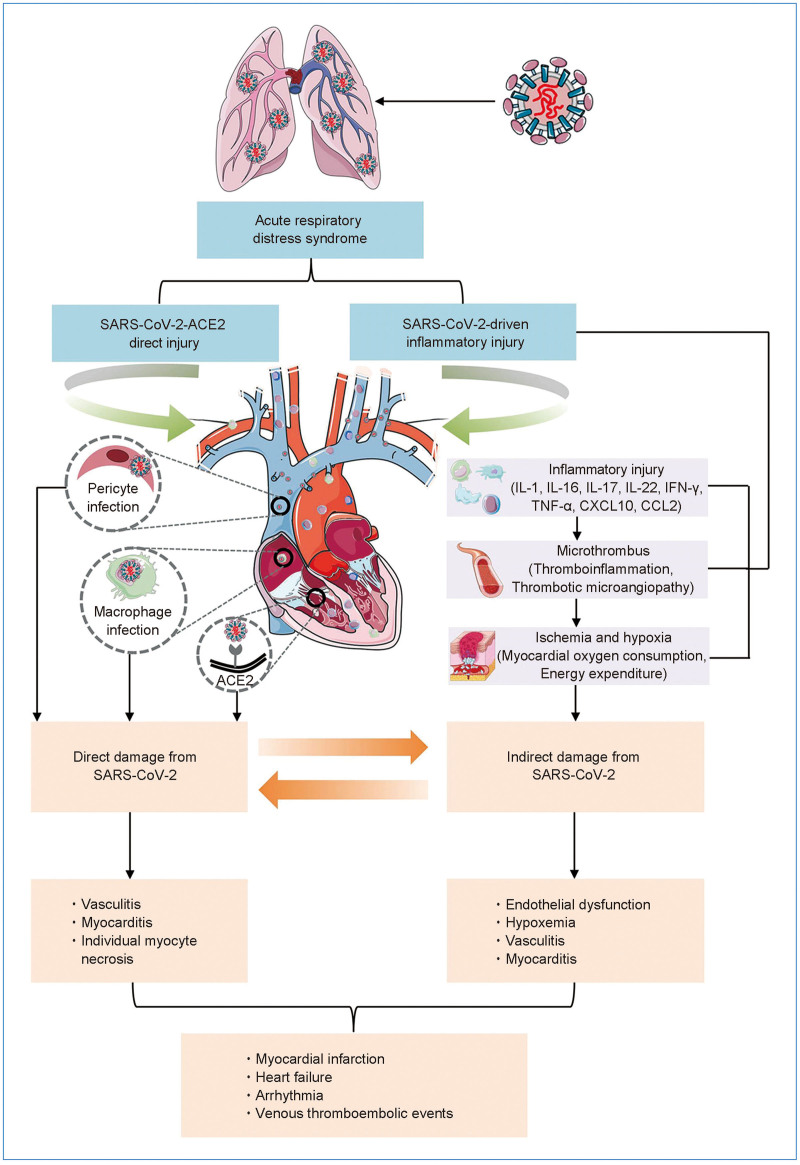
**Injury mechanism of cardiac complications caused by COVID-19.** ACE2: angiotensin converting enzyme 2; CCL2: C–C motif ligand 2; COVID-19: coronavirus disease 2019; CXCL10: C-X-C Motif Chemokine Ligand 10; IL-1: interferon 1; IL-16: interferon 16; IL-17: interferon 17; IL-22: interferon 22; IFN-γ: interferon γ; SARS-CoV-2: severe acute respiratory syndrome coronavirus 2; TNF-α: tumor necrosis factor α.

Interestingly, diffuse lymphocytic myocarditis was not discovered in patients with traditional viral myocarditis without COVID-19; cardiac histopathology studies have reported the expected absence of confluent myocyte necrosis in fulminant myocarditis^[20,22,51–53]^. Conversely, a greater number and more diffuse distribution of CD68^+^ cells has been reported in the heart tissues of patients with COVID-19, suggesting that the cells of monocyte/macrophage lineage may be dominant in the hearts of patients with COVID-19 compared with lymphocytes^[50]^. These findings indicate that COVID-19–induced myocarditis is different from the typical lymphocytic myocarditis with viral myocarditis presentations, suggesting that SARS-CoV-2–associated myocarditis may instead be correlated with diffusely infiltrative cells of the monocyte/macrophage lineage^[49,50,52]^. Based on the above, although there is no adequate evidence suggesting that SARS-CoV-2 directly infects myocardial cells, SARS-CoV-2 can lead to direct heart injury by reducing the expression of ACE2 or infecting interstitial cells (Figure 1).

## COVID-19–ASSOCIATED CYTOKINE STORM IN MYOCARDITIS

Previous studies have reported that the mechanisms underlying heart injuries in patients with COVID-19 are associated with the unusual inflammatory responses elicited by COVID-19. Approximately 7 to 10 days after COVID-19 onset, high cytokine levels are secreted, leading to a high inflammatory response in patients with COVID-19, which is probably the main cause of COVID-19 pneumonia and acute respiratory distress syndrome. Previous studies have demonstrated that proinflammatory cytokines such as IL-2, IL-6, IL-7, TNF-α, C-X-C motif cytokine 10 (CXCL10), and CCL2 are released into the circulation of patients with severe COVID-19 infection^[6,7]^, which is described as a cytokine release syndrome (CRS) and can overactivate T lymphocytes, resulting in myocardial damage. Among these cytokines, increased serum levels of IL-6 observed in CRS have been identified as a predictor of mortality and morbidity in retrospective studies of COVID-19^[6]^. This hyperinflammation could be the cause of acute heart failure and thromboembolic events^[54–58]^. The inflammatory activation and oxidative stress present in these patients may predispose them to a more severe clinical course following infection, leading to increased mortality of the patients with COVID-19 and heart failure^[59,60]^.

Moreover, the increased activation of innate immune pathways alongside the surge in proinflammatory cytokines, deregulation of thromboinflammation, thrombotic microangiopathy, and endothelial dysfunction can trigger an unbalanced host immune response, which is also revealed in the pathogenesis of COVID-19–related cardiac injury^[50,61]^. Thus, the types of cardiac inflammation caused by COVID-19 include extensive myocarditis, focal active myocarditis, multifocal myocarditis, and infiltrates with no myocyte damage. Infiltrates with no myocyte damage represent the highest amount of cardiac inflammation (44%).

Immunostaining revealed scattered CD4^+^ and CD8^+^ lymphocytes adjacent to the vascular structures. In uncommon locations, lymphocytes are adjacent to blood vessels while no peripheral degenerated myocytes are visible. The heart demonstrates a single-cell dropout/necrosis/apoptosis pattern, independent of any lymphocytic infiltration. Few CD3^+^ T cells can be found in the myocardium^[62]^. Macrophage density was superior in the epicardium and myocardium in the 2019 coronavirus disease group compared with the control group. Coronavirus 2019 disease hearts with single CM necrosis exhibited higher epicardial macrophage density compared with hearts without CM necrosis. These observations contribute to our growing understanding regarding the role of macrophages in the pathophysiological response to SARS-CoV-2 infection^[63]^. Most patients exhibit interstitial macrophage increase but show no associated CM damage. Basso *et al.*^[49]^ demonstrated that SARS-CoV-2-induced myocardial inflammation is predominantly mediated by macrophages. Nevertheless, some researchers consider that macrophage infiltration may reflect the underlying disease and not COVID-19.

Leng *et al.*^[30]^ detected myocardial cells and microvessels in myocardium infiltrated by lymphocytes and monocytes under pathological conditions, leading to myocarditis, individual myocyte necrosis, and vasculitis in the heart tissues of patients with COVID-19. Using the laser microdissection approach combined with proteomics, they discovered that the pathological changes in myocardium or microvessels due to the infiltration of the inflammatory cells and factors exhibited region specificity at the spatial level. Furthermore, they reported that multiple proteins involved in the inflammatory response of the high expression of inflammatory cells infiltrated the myocardium of patients with COVID-19, particularly the right atrium myocardia. Energy metabolism–associated proteins were downregulated in the myocardium of COVID-19 patients compared with that of patients without COVID-19. For instance, the left atrium–specific highly expressed protein translocase of inner mitochondrial membrane 50 (TIMM50) was significantly downregulated in the myocardia of patients with COVID-19 patients, which can result in heart failure and myocyte death. The researchers also reported that cardiac conduction, including calcium-mediated signaling, membrane potential, and cell communication–associated proteins, was downregulated in the myocardium of patients with COVID-19, which could be a cause of cardiac conduction abnormalities in COVID-19. Based on the above, a high inflammatory response in patients with COVID-19 can induce inflammatory cells infiltration into the myocardium and promote specific chamber–associated molecular signal changes, leading to heart failure (Figure 1).

## HYPOXIA-INDUCED CARDIOVASCULAR INJURY IN PATIENTS WITH COVID-19

Cytokine storm induced an increase in the levels of cytokines, such as IL-1, IL-16, IL-17, IL-22, IFN-γ, and TNF-α, which also played a role in myocardial injury by inducing endothelial dysfunction, platelets activation, neutrophils recruitment, and eventually a hypercoagulable state^[64,65]^. Patients with COVID-19 exhibit higher frequency and severity of clotting events along with increased D-dimer plasma, C-reactive protein, P-selectin, and fibrinogen levels^[66–68]^. Post-mortem fibrin microthrombi were even more common (80%) compared with acute ischemic injury (13%) and myocarditis (33%), suggesting that thrombosis was involved in accentuating myocardial injury, which is a major complication of COVID-19^[14,24]^. Microthrombus fibrin and terminal complement C5b-9 immunostaining results were similar to those of intramyocardial thromboembolic in patients without COVID-19. Microthrombosis can lead to endothelial dysfunction, which is considered a key contributor to the COVID-19 vasculopathy pathogenesis^[69–71]^. Leng *et al.*^[30]^ employed spatial region–resolved proteomics to conform whether the molecular function of microvessels in the myocardium infiltrated by monocytes or lymphocytes is damaged. They discovered that the proteins of acute inflammatory and innate immune responses were predominantly elevated in the cardiovascular system of patients with COVID-19, especially in the left atrium, indicating regionally specific injury, which is characteristic of microvessel damage following COVID-19 infection.

The mechanism of heart injury induced by microthrombosis is thus a question that needs to be addressed. It was previously established that microthrombosis can hinder the capillaries of vital target organs, including the heart, brain, and lungs, causing ischemia and hypoxia. Previous studies have also demonstrated that patients with COVID-19 exhibit myocardial injury triggered by demand ischemia and stress- and hypoxia-induced myocardial injury^[72]^. Proteins correlated with hypoxic response were detected in the right atrium, and their expression difference among the four chambers of the heart was the most predominant^[30]^. Hypoxia and pulmonary microvascular damage can lead to right heart stress and myocyte necrosis^[73]^. Furthermore, COVID-19 can elevate myocardial oxygen consumption and energy expenditure^[74]^. The two abovementioned factors lead to hypoxemia in patients with COVID-19, which is an additional hallmark of COVID-19 and is associated with enhanced oxidative stress alongside reactive oxygen species production, intracellular acidosis, mitochondrial damage, and cell death^[74–77]^. Baseline comorbidities, involving metabolic syndrome, hypertension, and cardiovascular disease may also play a role. Moreover, ACE2 controls the renin–angiotensin–aldosterone and kallikrein kinin systems, which may lead to a “kallikrein storm” during COVID-19 and increase the vascular permeability, inflammation, and fluid accumulation^[78,79]^. Cardiovascular damage can be further induced by local microvascular effects, endocarditis-related microthrombosis, and renin–angiotensin homeostasis^[80,81]^. Based on the above data, the cytokine storm in patients with COVID-19 causes microthrombosis that triggers hypoxia in myocardial cells, leading to vasculitis and myocardial injury (Figure 1).

## CONCLUSIONS

Extrapulmonary complications due to COVID-19 threaten the health of patients significantly. In particular, cardiac disease caused by myocardial and vascular injuries is one of the main causes of death. Compared with viral myocarditis, virus-driven inflammatory cell–induced myocarditis die to ischemia and hypoxia occupied a dominant position. Furthermore, myocarditis and vasculitis injury mechanisms caused by COVID-19 differ from those of common viral myocarditis, which may be a keystone for developing therapeutic targets for COVID-19–induced heart disease in the future. Understanding the injury mechanism underlying COVID-19–induced heart complications is also crucial for the current emergency treatment of patients infected with the Omicron variant. Finally, it can help clinicians effectively treat such patients based on specific conditions and reduce mortality.

## FUNDING

This work was supported by Chinese Academy of Medical Sciences (CAMS) Innovation Fund for Medical Sciences (No. 2023-I2M-3-002), National High Level Hospital Clinical Research Funding (No. 2022-PUMCH-A-022), the Emergency Key Program of Guangzhou Laboratory (No. EKPG21-32), and the COVID-19 project from the Army Medical University (No. 2020XGBD08).

## AUTHOR CONTRIBUTIONS

LL and XWB conceived the review. LL wrote the original paper. LL and XWB reviewed and edited the manuscript.

## CONFLICTS OF INTEREST STATEMENT

The authors declare that they have no financial conflict of interest with regard to the content of this manuscript.

## DATA SHARING STATEMENT

Data sharing not applicable to this article as no datasets were generated or analyzed during the current study.
